# Translational Medicine is developing in China: A new venue for collaboration

**DOI:** 10.1186/1479-5876-9-3

**Published:** 2011-01-04

**Authors:** Xiangdong Wang, Ena Wang, Francesco M Marincola

**Affiliations:** 1 Department of Respiratory Medicine, Biomedical Research Center, Fudan University Zhongshan Hospital, Shanghai, PR China; 2 Infectious Disease and Immunogenetics Section (IDIS) - Department of Transfusion Medicine, Clinical Center, National Institutes of Health, Bethesda, MD 20892. USA

## Abstract

Translational Medicine is an emerging area comprising multidisciplinary Research from basic sciences to medical applications well summarized by the Bench-to-Beside concept; this entails close collaboration between clinicians and basic scientists across institutes. We further clarified that Translational Medicine should be regarded as a two-way road: Bench-to-Bedside and Bedside-to-Bench, to complement testing of novel therapeutic strategies in humans with feedback understanding of how they respond to them. It is, therefore, critical and important to define and promote Translational Medicine among clinicians, basic Researchers, biotechnologists, politicians, ethicists, sociologists, investors and coordinate these efforts among different Countries, fostering aspects germane only to this type of Research such as, as recently discussed, biotechnology entrepreneurship. Translational Medicine as an inter-disciplinary science is developing rapidly and widely and, in this article, we will place a special emphasis on China.

## The development of Translational Medicine in China

Translational Medicine is an emerging area comprising multidisciplinary Research from basic sciences to medical applications well summarized by the Bench-to-Beside concept; this entails close collaboration between clinicians and basic scientists across institutes. We further clarified that Translational Medicine should be regarded as a two-way road: Bench-to-Bedside and Bedside-to-Bench [1], to complement testing of novel therapeutic strategies in humans with feedback understanding of how human react to the treatment. It is, therefore, critical and important to define and promote Translational Medicine among clinicians, basic Researchers, biotechnologists, politicians, ethicists, sociologists, investors and coordinate these efforts among different Countries [2] fostering aspects germane only to this type of Research such as, as recently discussed, biotechnology entrepreneurship [3]. Moreover, the recognized need to base biomedical discoveries on knowledge derived from human samples should be covered by the development of high quality Biobanks [4] and tools for data mining of existing information [5]. Translational Medicine as an inter-disciplinary science is developing rapidly and widely and, in this article, we will place a special emphasis on China.

A first National step toward the promotion of Translational Medicine in China was to hold the first Symposium on Translational Medicine in 2007. Another milestone emphasizing the commitment of this Country to the rapid development of Translational Medicine was the Sino-America Symposium on Clinical and Translational Research co-organized by the GlobalMD Organization, Chinese Academy of Medical Sciences and the U.S. National Institutes of Health Clinical Center in June of 2010 [6]. The meeting aimed at gathering clinicians, Researchers, ethicists and health care officials from hospitals, academia and governmental agencies, involved in human subject Research, multi-national clinical trials, and Translational "bench-to-bedside" implementation of Research that apples broadly accepted ethical regulations for quality Research. A number of important themes relevant to bilateral collaborations between the USA and China were discussed, e.g. current status and environment of clinical and Translational Research in the U.S. and China, perspectives and new directions in global health Research, bioethics of drug trials and human subjects Research protection, drug trials and drug development strategies, approaches to the study of rare diseases and its benefit to the broader clinical community, the study of emerging infections, gene therapy and genomics--genetic and cell-based technologies, early diagnosis and prevention of heart disease, clinical and Translational Research in oncology, and stem cell therapeutic potential. During the first six months of 2010, at least seven Institutes or Centers for Translational Medicine were established in China; among them the Union Center for Translational Medicine can be considered a further milestone toward the development of Translational Medicine in China as pronounced by Professor Zhu Chen, Minister of Ministry of Health of China.

Currently, Translational Medicine in China is focused predominantly on cancer, acute and chronic diseases, common and widespread infections. Cancer in particular is addressed not only at the primary stage but increasingly as a systemic disease whose diagnosis, prognosis and prediction of responsiveness to therapy needs to be best assessed through the development and validation of reliable biomarkers [7]. A focus of present funding is the design of integrated strategies for combination therapies that could embrace treatments of complex diseases such as cancer from different aspects of their biology simultaneously as recently discussed by Ascierto et al. [8]. In this regard, the *Journal of Translational Medicine *in about to launch a new sub-section dedicated to the subject of combinatorial therapies and the scientific, regulatory and financial hurdles associated with this approach. Moreover, emphasis will be placed on the development of targeted and personalized therapies aimed at treating patients and their disease according to our modern understanding of their genetics. These novel approaches aimed at treating patients at the early stage, with advanced diagnostics based on cutting edge technologies were recently discussed among preclinical and clinical scientists, representing academia, industry, China Government and other Countries at the Symposium for Advanced Biotechnologies & Instrumentations held in Shanghai in October of 2010 [9]. Topics included application of antibody microarrays to develop disease-specific diagnostics for the prediction and indication of disease duration, severity, response to therapies and prognosis. Efficient prevention and therapy for common and serious infectious diseases attracted great attention from both national opinion leaders and politicians. It was emphasized that methodologies and experiments related to such diseases should be efficiently translated into clinical practice. Reliable, cost-efficient biotechnologies aimed at prevention and/or early diagnosis of disease should be encouraged. Correspondently, the number of Biomedical Science Parks has been growing in China, through which it is expected to increase the commercial development of biomedical and biotechnological products. These Science Parks provide special opportunities, e.g. financial and administrative support, appropriate facilities and priority policies for Translational Medicine [10].

## Financial Commitment and Sources

A challenge for the effective development of Translational Medicine in China is the need to finance sufficiently new and developing areas of investigation. One of the largest sources of financial support is the National Nature Science Foundation of China who has approved a 90 billion RMB (13.5 billion USD) allocation for the 2010 fiscal year focusing on projects with potential clinical applications. Drug discovery and development is expected to support strong economic growth within the Country and globally. Projects and applications with potential for clinical usefulness and benefits to patients are strongly encouraged and prioritized over more speculative projects. Moreover, specialized foundations initiated by experts in a particular Research/clinical area are emerging intended to support specifically some fields of Research such as the Beijing Lishen Cardiovascular Health Foundation [11]. Other private foundations of broader breath also play an important role in the development of Translational Medicine in China, e.g. Tang Foundations provided 100 million RMB (15 million USD) to establish the new institute for Translational Medicine in Jiaotong University. The primary mission of the Tang Foundations is to support education, healthcare, and community service as bridging efforts and resources between American and Chinese entities [12]. Several Universities are also becoming increasingly interested in supporting Translational efforts and several collaborate with the local governments and/or companies to create new centers for Translational Medicine. On those lines, the first hospital of Wen Zhou Medical College is actively organizing the International Conference of Translational Medicine 2011 under the auspices of the new International Society for Translational Medicine [13].

## Remaining challenges to Translational efforts

As in other countries, several challenges need to be recognized and overcome. Among them is the clarification of the definition of Translational Medicine [14]. Moreover, a better alignment of the goals of Translational Medicine with the incentives motivating individual scientists' work need to be achieved [15,16]. It will be helpful to establish international and standardized criteria for the evaluation of the goals and successes of Translational Medicine keeping in mind that although often overlapping basic scientific Research differs from Translational Medicine for the direct applicable potential of the latter. It should be bore in mind that Translational Medicine is not a "magic word" covering all aspects of sciences but rather a tool to enhance the efficiency in which science is performed by integrating areas of expertise through a broad spectrum of disciplines [2]. Moreover, it could be argued that Translational Science/Research and Translational Medicine may represent two distinct aspects of the "translation" process. For example, the concept of Translational Medicine has been well-accepted by the pharmaceutical sector that recently established Drug Discovery World, a new organization responsible for Translational Medicine [17] with emphasis on how to efficiently translate ideas into sustainable projects through the identification of criteria for rapid validation in humans of dose schedules and strategies of administrations tested in animals. The program also focused on identification of surrogate biomarkers that could test in the short-term drug efficacy decreasing the length and cost of extensive phase III clinical trials or at least providing better information about the rational to embark into any of them. Thus, the definition of Translational Medicine may be different for these stake holders compared to Academia and Government who may be more interested in broader and more general attempts to identify novel therapeutic strategies through direct human observation; a goal that could be better encompassed by the term "Translational Science/Research". Yet, it needs to be kept in mind that, although "Translational" may mean different things to different stake holders, the overall goals are similar, overlapping and not mutually exclusive; recognition of the diversity of meaning is helpful to understand each other but should not be considered a barrier to a synergistic relationship among those interested in fostering the development of Research for the benefit of the ill [2]. It is hopeful that efforts to congregate distinct participants to the Translational process into a society devoted to the efficient exchange of information such as the newly instituted International Society for Translational Medicine [13] may ultimately yield the expected results with the required efficiency.

## Enhancing communication through broad reaching yet specialized editing

It may be difficult to balance the need to reach a broad audience among disciplines while maintaining a high quality peer review process; for this reason, the *Journal of Translational Medicine *has developed specialized subsections whose editorial board has both a broad-based interest for Translational Medicine and expertise in specific areas relevant to the discipline [18-20]; additional subsections are in the making not necessarily dedicated to a specific Research area rather to problematic concepts common to multiple fields such as the development of combinatorial therapies, efficient clinical testing a nd drug development or science policy analysis. These subsections attempt to address specific areas of broad interest almost as a task force created *ad hoc *and aimed at identifying solutions to specific problems [21]. Similar subsections could be created for the discussion of issues relevant to Translational Medicine but of specific relevance to China. Moreover, awards could be proposed to provide incentive to young investigators willing to embrace the hurdles of translational disciplines [22]; press-releases or other forms of public communications that could help bridge the divide between science and journalism can be incrementally implemented to enhance public awareness and support for translational efforts [23]. Finally, rapid publication of task force-based analyses about issues relevant to Translational Research will enhance the usefulness of efforts by individual Organizations and/or Countries addressing global problems as recently exemplified by the International Society for the Biological Therapy of Cancer task force on biomarker discovery [24-26]. Such focused efforts addressing areas of broad interest while emerging from the Chinese community, facing Chinese challenges and providing Chinese solutions will prove invaluable for the growth of the global Translational Medicine community.

## Conclusions

There is a potential for great future impact on the national economical growth that could be generated from newly established centers and/or institutes for Translational Medicine in China. This will largely depend on collaboration between China and other Countries, sharing the understanding, methodologies, Research protocols and resources, and development. The International Conference on Translational Medicine (ICTM 2011) to be held in WenZhou, China in 2011 will be an opportunity for Chinese scientists and Researchers to communicate and introduce their developments and strategies to international experts [27]. Global opinion leaders and institutes/centers on Translational Medicine are warmly welcome to share opportunities and combine efforts to resolve challenges that face the development of Translational Medicine in China and/or the World, by establishing Research projects, organizing educational programs, applying for Research grants.

## Competing interests

The authors declare that they have no competing interests.

## Authors' contributions

XW collected the salient information about Chinese Translational Medicine Efforts in the last decade, EW and FMM contributed a general overview of the field of Translational Medicine and integrated the information about Chinese data with the broader scope of the *Journal of Translational Medicine*. All authors read and approved the final manuscript.
